# Effect of Electrospinning Network Instead of Polymer Network on the Properties of PDLCs

**DOI:** 10.3390/molecules28083372

**Published:** 2023-04-11

**Authors:** Yuzhen Zhao, Tingting Lang, Chaonian Li, Wenbo Yin, Yitian Sun, Ruijuan Yao, Cheng Ma, Zuhui Shi, Dong Wang, Zongcheng Miao

**Affiliations:** 1 Xi’an Key Laboratory of Advanced Photo-Electronics Materials and Energy Conversion Device, School of Electronic Information, Xijing University, Xi’an 710123, China; zyz19870226@163.com (Y.Z.); langtingting516@163.com (T.L.); lichaonian0513@126.com (C.L.); ywb1849801751@163.com (W.Y.); sunyitian0524@163.com (Y.S.); yaoruijuan0223@163.com (R.Y.); mcjd1103@126.com (C.M.); 2 Department of Materials Physics and Chemistry, School of Materials Science and Engineering, University of Science and Technology Beijing, Beijing 100083, China; hankszh@163.com; 3 School of Artificial Intelligence, Optics and Electronics (iOPEN), Northwestern Polytechnical University, Xi’an 710072, China

**Keywords:** polymer-dispersed liquid crystals, electrostatic spinning, nanofiber doping, electro-optical properties

## Abstract

In this study, polymer-dispersed liquid crystal (PDLC) membranes were prepared by combining prepolymer, liquid crystal, and nanofiber mesh membranes under UV irradiation. EM, POM, and electro-optic curves were then used to examine the modified polymer network structure and the electro-optical properties of these samples. As a result, the PDLCs with a specific amount of reticular nanofiber films had considerably improved electro-optical characteristics and antiaging capabilities. The advancement of PDLC incorporated with reticulated nanofiber films, which exhibited a faster response time and superior electro-optical properties, would greatly enhance the technological application prospects of PDLC-based smart windows, displays, power storage, and flexible gadgets.

## 1. Introduction

Films with polymer-dispersed liquid crystals (PDLCs) are made of LC molecules encapsulated in a polymeric base material. Due to the LC molecules’ random orientation in the polymer network, when there is no electricity, the PDLC film presents a diffuse white scattering fog state [1,2,3]. This is because the LC droplet is neatly deflected along the direction of the electric field, matching the polymer network’s index of refraction n_p_ with the universal index of refraction n_o_ of LC [4,5,6,7,8,9]. The film changes from a white scattering condition to a highly transmissive state when a sufficiently high voltage is applied. Due to the amazing quality of PDLCs, significant progress has been made in the study of these components over the past decade. Consequently, they are now in widespread use during quantum dot (QD) films [10,11], organic light-emitting diodes (OLEDs) [12], field-effect transistors (FETs) [13,14], energy storage [15], and solar harvesting elements [16].

With the development of research, enhancing the holistic achievement of PDLC films has become a major research issue, especially in terms of improving photovoltaic performance and increasing durability. In previous studies, the doping of nanomaterials, addition of dyes, adjustment of polymerization requirements, and modification of polymer matrix microstructures have led to excellent electro-optical properties of PDLCs [17,18,19,20]. To create PDLC films, Lu et al. [21] created a wavelength-selective, two-stage polymerization of acrylate-thiol. A PDLC with a microsphere polymer shape has been successfully created using this method, which significantly increased the contrast ratio (CR) and adhesion without compromising any other features. The effects of light initiator concentrations on the morphology and electro-optical characteristics of PDLC films with thiol and acrylate moieties were studied by Nasir et al. By maximizing the photo initiator content and maintaining stable LC and monomer concentrations, PDLC films were created that have good transmittance and little switch time, haze, or power usage [22,23]. At a low driving voltage, the optimized samples can obtain ΔT (difference between on-state and off-state transmittance) >85%.

However, issues, such as responsiveness, driving voltage, off-state transmission rate, and durability, are still the focus of research on PDLC films under conventional processes, and these properties greatly limit its application scope. To improve the electro-optical characteristics of PDLC films, polymer networks must be created and interactions between polymer networks and liquid crystal droplets must be managed. Consequently, electrostatic spinning films were added to PDLCs to enhance the holistic achievement of PDLC films [24,25,26,27]. In order to create filamentary fibers at the nanoscale scale, electrostatic spinning can be used. This results in high-porosity and high specific-area effects that have a range of applications in the optoelectronics industry [28,29,30,31]. The excellent combination of PMMA nanofiber films with superior optical transmittance in the visual range, high ionic conductivity, excellent UV resistance, and good physicochemical stability allows a realistic approach to this concept. This study was conducted to design a mesh-like nanofiber PDLC film with consistent thickness and high light transmission using electrostatic spinning technology. Compared with the conventional preparation methodology, the PDLC films made from nanofibers, prepolymers, and liquid crystals have the advantages of consistent composition density, well film forming property, stable structure, and superior electro-optical properties. By contrasting the adjustments in the polymer network morphology and electro-optical shapes, the effect of reticular PMMA nanofibers on PDLC films was investigated, and the problems of poor robustness and slowness were solved.

## 2. Results and Discussions

### 2.1. Property Changes in PDLC Films as a Result of Liquid Crystal Content

**Figure 1:**
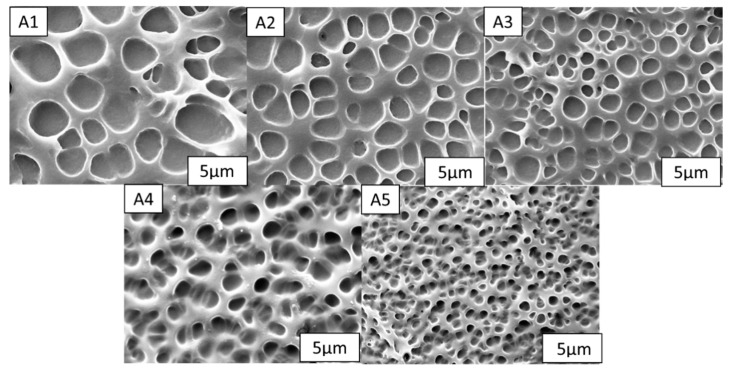
Network of the polymer matrices in specimens (**A1**–**A5**).

Liquid crystals have an important role in PDLC constructions because they are dependent on the applied voltage to control the electro-optic axis orientation of liquid crystal droplets and adjust the liquid crystal particles’ refraction index to match the polymer matrix. The composition of the liquid crystals used to create these films has a significant impact on their electrical properties; if the liquid crystal content is too low or too high, a PDLC structure will not form. Transmittance, threshold and saturation voltages, and contrast are all significantly affected by the structure and the molecular weight of polymer, the morphology of LC drops in a polymeric matrix, and its properties, in particular, the chemical composition [32,33,34,35]. Investigating how the liquid crystal concentration influences aspects of PDLC films that are photoelectric is critical. Controlling the structure of the polymer is significantly influenced by the quantity of liquid crystals present. Figure 1 displays SEM images of the liquid crystal polymer network vastly differing in structure. As the liquid crystal content decreases from 90% to 40%, samples A1 and A5 show a reduction in the size of the pores in the porous structure. In other words, when the liquid crystal concentration increased, the UV polymerization process separated the phases more thoroughly, the matrix density decreased, and the network size increased.

**Figure 2:**
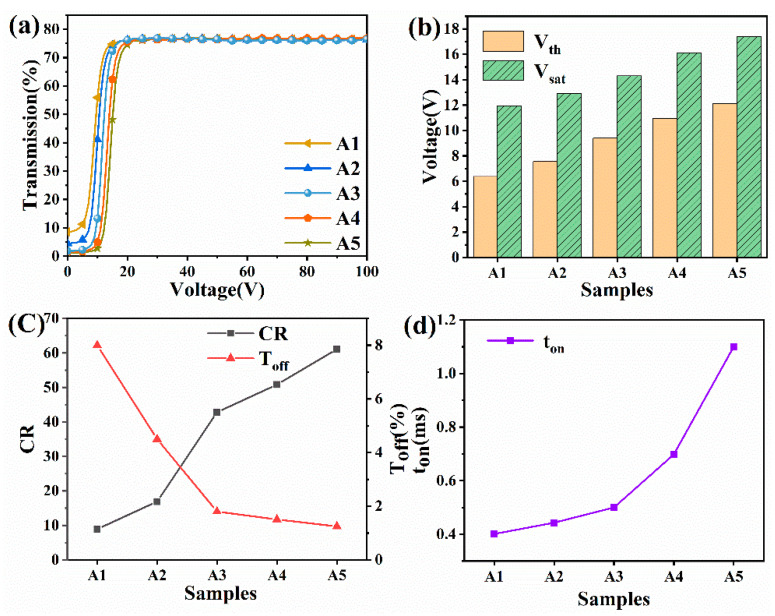
(**a**) Reliance of the transmittance of specimens A1–A5 upon that applied electric field (100 Hz); (**b**) Saturation voltage and threshold voltage of specimens A1–A5; (**c**) Contrast ratio and off-state transmittance of specimens A1–A5; (**d**) Reaction speed of specimens A1–A5.

A thorough liquid crystal parametric device was used to examine the electro-optical characteristics of specimens in Team A. The findings demonstrate that the electro-optical characteristics of PDLC films are remarkably influenced by the liquid crystal content. According to Figure 2a, the electro-optical property of various liquid crystal compositions varied greatly; samples A1–A5 show an increase in light transmission with voltage. For samples A1 through A5, Figure 2b depicts the increasing trend of the threshold and saturation voltages as the SEM result of the decrease in the polymer network size from samples A1 to A5 (Figure 1). As a result, the driving voltage needed for such reorientation of liquid crystal molecules and the surface anchorage force of the polymer network both increase progressively. Figure 2c illustrates the T_off_ and CR fluctuation bights for such Team A specimens. The findings demonstrate that the transmittance of samples A1 to A5 drops, while their contrast increases in the closed state. Due to the system’s higher polymer content and viscosity, the system’s polymerization reaction rate accelerates and the extent of the polymerization network shrinks, increasing the specific surface area of the liquid crystal droplets and the quantity of contacts between the droplets and cavities. Because incoming light irradiates the PDLC film near the interface, where there is considerable scattering and refraction, the sample’s contrast in the off-state decreases and its transmittance increases. T_on_ is seen to gradually grow from samples A1 to A5 in Figure 2d. The increase in flexure power there at the interface of the polymer network and the droplet is caused by the decrease in cavity size and the increase in specific surface area of the polymer network. It takes some time for the liquid crystal particles to rapidly return to their original state after the voltage is removed, and as such the tons are increased progressively.

### 2.2. Effect of the Thickness of the Nanofibers on the Performance of PDLC

**Figure 3:**
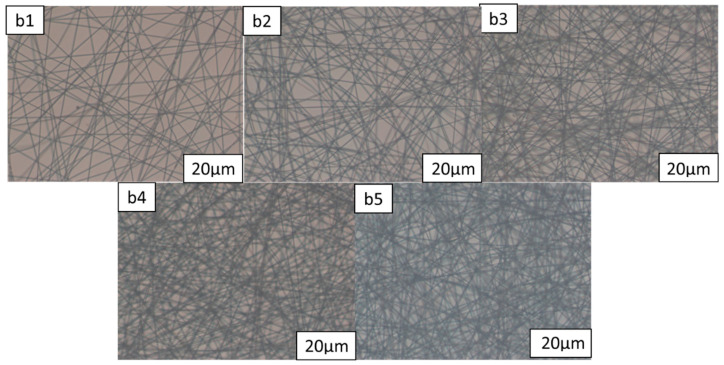
Polarized optical microscopy (POM) of nanofibers of (**b1**–**b5**).

**Figure 4:**
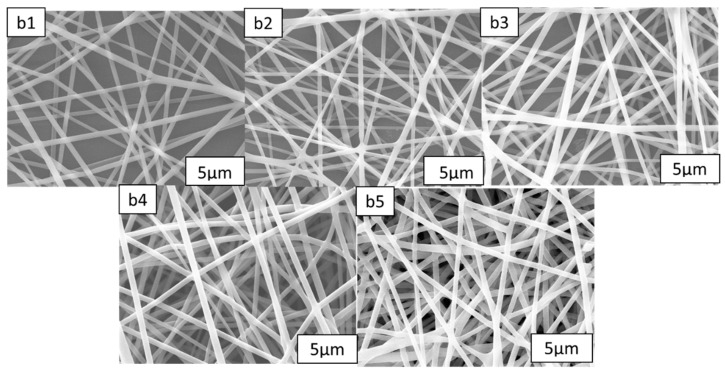
Microscopic morphology of nanofibers of (**b1**–**b5**).

Using PDLC systems with the same composition, different mesh nanofiber film thicknesses were added, polymerized, and tested to see how they affected the optoelectronic characteristics of the material. The DMF solution and PMMA solute were thoroughly mixed before being homogenized using a sonicator. By adjusting the electrostatic spinning time to the precise formulations, the thickness of the PMMA nanofiber films was managed. Table 1 lists the formulations b1–b5. Following the procedures in Section 3.2, nanofiber films of various thicknesses were created and subsequently characterized using polarized light microscopy (POM), as shown in Figure 3. The microscopic morphology of samples b1–b5 was investigated by scanning electron microscopy after the application of gold spray, as shown in Figure 4. Figure 3 and Figure 4 show that the nanofibers’ morphology remained largely unchanged as the electrostatic spinning time increased and they were able to spin fibers with uniform diameters and without beads. As the nanofiber film samples b1–b5 became thicker, their porosity and specific surface areas increased as well.

**Figure 5:**
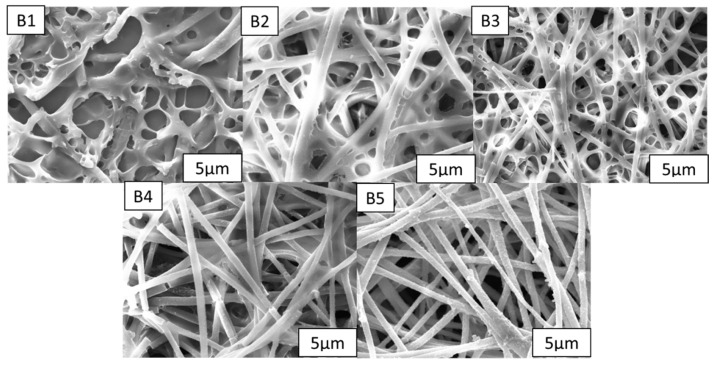
Network of the polymer matrix of (**B1**–**B5**).

The polymer network morphology for samples B1–B5 is depicted in Figure 5. As the nanofibers are thickened, they have an obvious impact on the polymerization reaction. Figure 5 shows that as the nanofiber film is thickened, the polymer network becomes less obvious, indicating that the polymerized products have been dissolved. For the B5 sample, almost no polymer network has been observed here. The same liquid crystal blend formulations and polymerization conditions were used for samples B1 through B5; therefore, only the thickness of the nanofiber film may be used to explain changes to the polymer microstructure. The primary cause of the shift in LC microdrop size, according to the SEM results, is, therefore, the thickening of the nanofibers, which increases their specific surface area and porosity and leads to a smaller polymer mesh.

**Figure 6:**
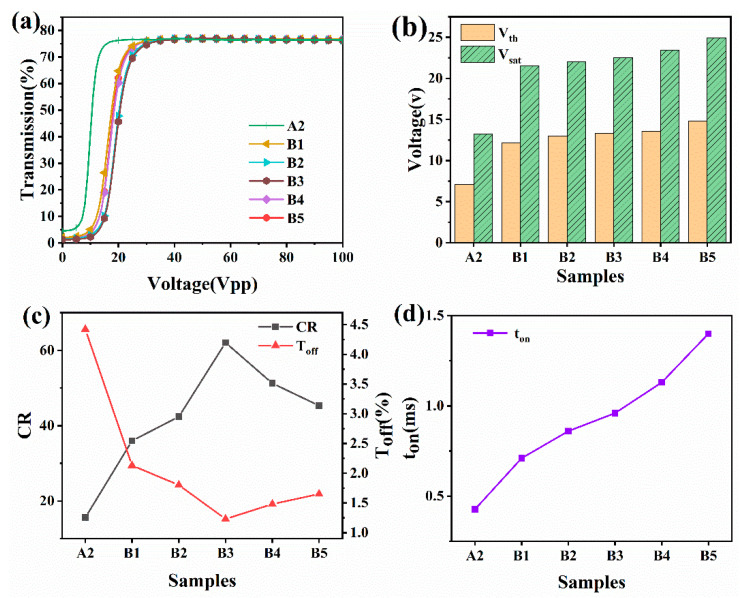
(**a**) Species’ transmittance varies depending on A2 and B1–B5 on the applied electric field (100 Hz); (**b**) off-state emissivity and comparison ratio of recordings A2 and B1–B5’s; (**c**) saturation and threshold voltages for recordings A2 and B1–B5’s; and (**d**) reaction times for recordings A2 and B1 through B5.

This series of tests looked at how the electro-optical characteristics of the same PDLC system changed when it was loaded with nanofiber films of various densities, while A2 was not. The electro-optical curves for the same liquid crystal blend formulation and the identical polymerization conditions of PDLC loaded with various nanofiber film thicknesses are shown in Figure 6a. This model shows that adding various mesh nanofiber film thicknesses had no impact on the fundamental properties of the PDLC films and that it is still possible to toggle between completely transparent and opaque films. The necessary threshold and saturation voltages anticipated to witness significant growth are visualized in Figure 6b. The preliminary findings indicate that the thickness of the loaded nanofibers increases the saturation voltage. For specimen B5, the threshold voltage increases to 14 V, and the saturation voltage increases to 25 V. This is because the gradual contraction of the polymer network’s cavity and the increase in nanofiber porosity, which causes a larger overall crystalline functionality and a higher voltage required to achieve diversion after energization. Figure 6c demonstrates the T_off_ and CR curvatures for the specimens from groups A2 and B1–B5. The samples in groups B1–B5 had lower T_off_ values and significantly higher CR values, especially in comparison to specimens from cluster A. The largest influences on the T_off_ of the PDLC films are the network morphological traits and polymer’s optical properties. The PDLC fiberglass had higher development at more interfaces between the LC liquid and the various stages as a result of the addition of nanofiber films of different thicknesses. This improved the PDLC films’ capacity to scatter incident light and decrease the T_off_ values. Lower T_off_ values are also a consequence of such nanofiber films’ lowered ability to match the refracting powers of the LC drops and the entire polymer network. Figure 4b1–b5, show that the polymer network contracts, the porosity increases, and the liquid crystal droplets enlarge. Figure 6 depicts what happens whenever the voltage is turned on: the anchoring force increases, the ability of the liquid crystal particles to react quickly decreases, and the t_on_ increases (Figure 6d).

### 2.3. Effect of Identical Nanofiber Membranes on PDLC’s Photovoltaic Characteristics with Different Ratios

**Figure 7:**
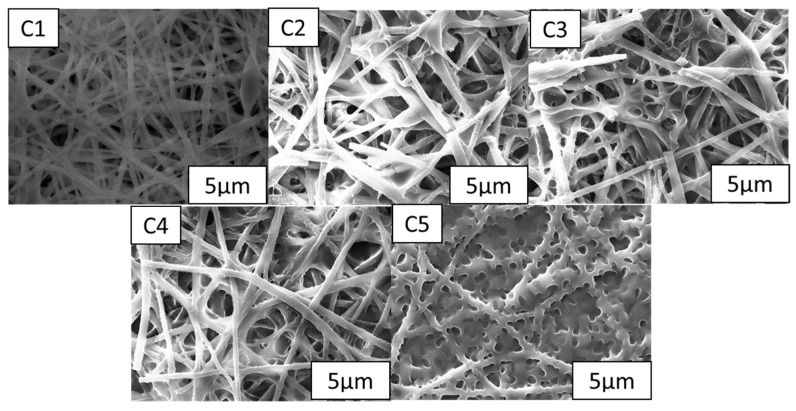
Network of the polymer matrices in specimens (**C1**–**C5**).

In the series of studies to examine the impact of nanofiber thickness in regards to the electro-optical characteristics of PDLC films, mesh nanofibers were added to PDLC films with varying ratios under the same electrostatic spinning circumstances (reference b3). The polymer network’s morphological characteristics created by samples C1–C5 while spinning at b3 circumstances is depicted in Figure 7. There was a significant influence of the introduction of reticulated nanofiber films on the PDLC polymer network formation in different proportions. With the same nanofiber film, as the percentage of prepolymer gradually increases from C1 to C5, the diameter of the mesh decreases, the drops in liquid crystal become more numerous, the size decreases, and the specific surface area increases.

**Figure 8:**
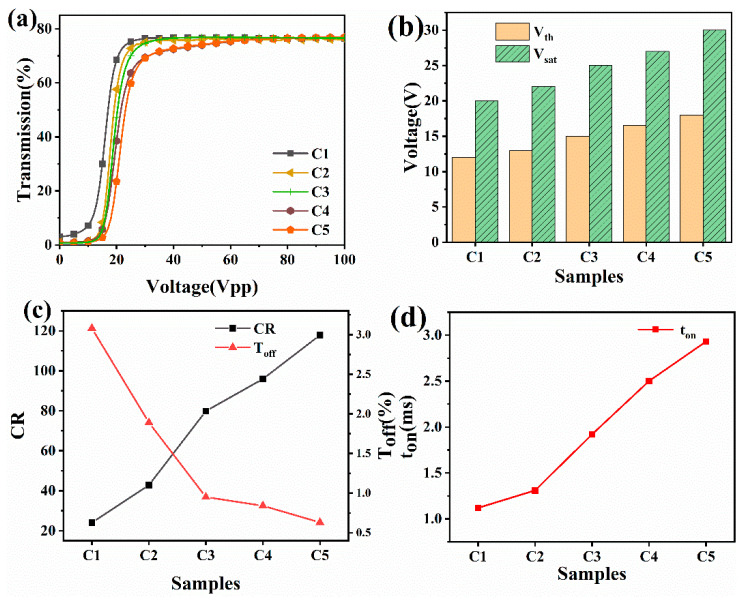
(**a**) Transmittance of specimens C1–C5 is dependent on the applied electric field (100 Hz); (**b**) Contrast ratio and off-state transmittance of specimens C1–C5; (**c**) Threshold voltage and saturation voltage of specimens C1–C5; (**d**) Reaction times of specimens C1–C5.

Figure 8a is the electro-optical bights of group C under different liquid crystal blend formulations at the same polymerization conditions. One can see from the figure that the basic properties of the PDLC films are not changed by the nanofiber films, and the change from fully transparent to opaque is still possible, with the electro-optical curves of C1–C5 shifted to the lower right. According to the findings in Figure 8b, the prepared specimens’ threshold and saturation voltages increase as the prepolymer content does. The threshold voltage of sample C5 was found to increase by 6 V compared with A5. This is primarily caused by the polymer network’s unit space volume decreasing and its unit space volume increasing, which leads to the overall larger liquid crystal space structure interface and the increase in voltage after energization to complete the deflection. The results in Figure 8c show that when compared to the specimens in cluster A, the specimens in clusters C1–C5 exhibit a significant reduction in T_off_ values and a boost in CR values. The T_off_ worth of the PDLC films is significantly influenced by the network form and complete index of refraction of the polymer. An increase in the quantity of LC droplets was seen in the new composite system as a result of the nanofiber inclusion, causing an enhancement in the number of networks and a consequent decrease in T_off_. Additionally, the altered phase interface and resulting reduction in T_off_ allowed the nanofibers to lower the index of refraction match between the polymer network and the LC microdroplets. Accordingly, the CR of C5 far exceeds that of the A5 samples compared to A5. The SEM images in Figure 7 show that the prepolymer mesh shrinks and the porosity increases as the polymeric monomer content increases in group C samples, and the narrowed LC microdroplets are subjected to increased anchoring forces when exposed to voltage, slowing down the reaction time of the molecular orientation transition, i.e., t_on_ increases, as seen in Figure 8d.

**Figure 9:**
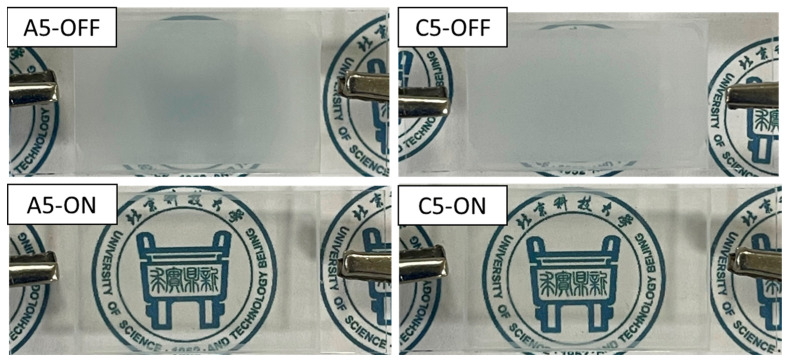
Actual pictures of samples A5 and C5.

The difference between the A5 and C5 samples is the presence or lack of nanofiber film doping. A physical plot of specimens A5 and C5 in both the on- and then the off-states are displayed in Figure 9, graphically demonstrating how the transmittance changes. The graphs show that the C5 sample is more transparent when it is in the on-state and has a lower light transmission when it is in the off-state. As a result, C5’s CR is superior to A5 due to the expansion of C5 in both phases, further demonstrating the elevated practical significance of nanofiber-doped PDLC substances.

### 2.4. High Temperature and Humidity Tests

**Figure 10:**
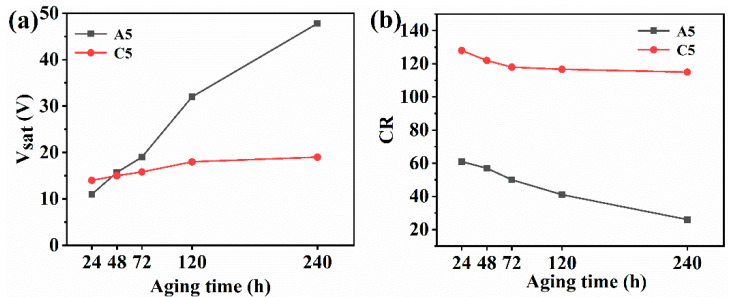
Environmental impacts of extreme air temperature, high relative dampness, and Ultraviolet light on electro-optical efficiency: (**a**) saturation voltage and (**b**) contrast ratio.

Samples A5 and C5 (doped with nanofibers) were exposed to UV light (365 nm), high temperature, and humidity (60 °C, 95% RH) for aging resistance measurements. Figure 10 shows that the V_sat_ of both types of films increases and the CRt decreases after long-term exposure to this environment. It is crucial to remember that the decline in the CR of A5 is greater than that of C5, while the increase in the V_sat_ of C5 is less than that of A5. This suggests that PDLC films doped with nanofibers perform more effectively than conventional PDLC films in extreme conditions and exhibit greater durability. PMMA nanofibers, based on promising physical and chemical properties, not only enhance the electro-optical properties of PDLCs, but also render the PDLC films significantly more stable, which provides unlimited possibilities for future development applications.

## 3. Experimental Section

### 3.1. Materials

The nematic LC was SLC 1717 (Shijiazhuang Chengzhi Yong Hua Display Materials Co. Ltd., Shijiazhuang, China; TN-I = 365.2 K; no = 1.519; ne = 1.720). UV 6301 was the polymer matrix utilized (Hanrui Industrial Co. Ltd., Shanghai, China). The chemical compound used was PMMA (500,000 g/mol) (Anhui Zesheng Science Co., Ltd., Hefei, China). Irgacure 651 served as the elicitor (Ciba Geigy, Jingjiang Hongtai Chemical Engineering Co., Ltd., Jingjiang, China). Sinopharm Chemical Reagent Co. sold dimethylformamide (DMF), which was purchased.

### 3.2. Sample Preparation

In the experiments, a solution of polymethyl methacrylate (PMMA) was added to dimethylformamide (DMF) solvent at 25% and agitated for 24 h at room temperature. Subsequently, with a pulse amplitude of 30%, its solution was dispersed by ultrasound for 60 min. The above solution was fed into a syringe fitted with a metal needle (20G) and placed in a nanoelectrostatic spinning machine (Tongli Micro-Nano Technology Co., Ltd., Shenzhen, China) with electrospinning flow rate and applied voltage set to 3.0 mL h^−1^ and 30 kV, respectively, in addition to a distance of 25 cm between the needle and aluminum foil. The environment for the manufacture of nanofiber films was maintained at 60% humidity at 25 °C spinning temperature. Transparent conductive glass and aluminum foil were attached and formed aggregation devices in the grounded state, and the electrospinning time was used to adjust the film thickness. The elements of the electrospinning step samples are depicted in Table 1. After completion of the electrostatic spinning process, the cavity liquid crystal cassette was fabricated from the attached spun conductive glass placed opposite to another piece of glass, in which the spacing was obtained from a 20 μm polyester film. The preparation of the liquid crystal cassette with loaded nanofiber film was finally completed.

**Figure 11:**
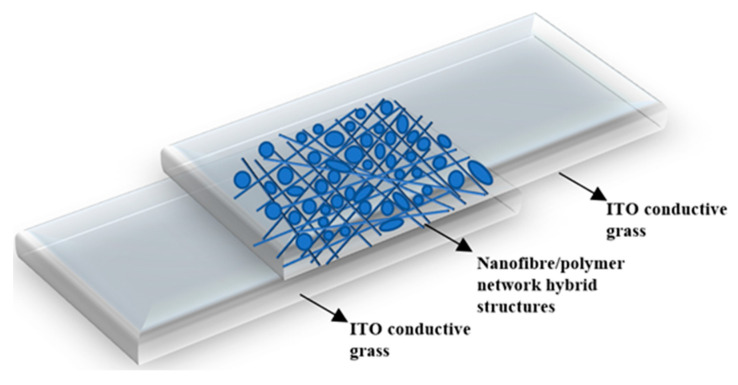
The structure of liquid crystal cell after polymerization.

To prepare PDLC films, mixtures of different ratios of monomer (UV6301)/initiator (IRG651)/LC (SLC1717) were shaken, mixed, and sonicated to obtain a homogeneous phase, and the ratio of the IRG651 in samples A-C series was 0.5 wt.% of the total mixture. Then, the well-mixed samples (compositions are shown in Table 2) were guided directly into the readied liquid crystal cassettes through capillary action. Finally, samples were placed at room temperature (25 °C) and under 365 nm UV light atmosphere for polymerization for about 10 min (UV light intensity is W = 10 mW/cm^2^). Figure 11 illustrates the structure of a polymerized liquid crystal cassette.

### 3.3. Methods of Characterization

The curve indicating the electro-optical property of PDLC films is among the most significant property index for thin-film optoelectronic devices. The contrast value, time–response curve, and transmittance–voltage curve (also known as photoelectric curve) of PDLC films can all be precisely measured using the LCD parameter tester (LCT-5016). The photoelectric characteristic test was used to determine the threshold voltage (*V_th_*), drive voltage (*V_sat_*), contrast (CR), off-state transmittance (T_off_), off-state response time (t_on_), and other parameters. When the PDLC film achieves a 10% T_on_ effect, the voltage is known as the threshold voltage (*V_th_*). By balancing surface contacts, applied electric forces, and elastic forces, the theory of threshold voltage is mathematically represented, leading to the following conclusions:

(1) $$V_{th}=\frac{d}{3a}\left[\frac{\rho_{p}}{\rho_{LC}}+2\right]{\left[\frac{k\left(l^{2}-1\right)}{\Delta \varepsilon }\right]}^{\frac{1}{2}}$$

where d is the film thickness, $\rho_{p}$ is the polymer’s resistivity, $\rho_{LC}$ is the liquid crystal’s resistivity, and the ratio of sizes l=a/b where the long and small axis lengths of liquid crystal droplets are a and b, respectively. The dielectric anisotropy and the elastic constant are k and Δε, respectively. With further increase in voltage, increasingly more LCs are aligned with the magnetic field. The LC droplet could overcome the polymer–liquid crystal interfacial forces when it reaches its minimum free energy [36], during which time the LC molecules are perfectly parallel to the orientation of the magnetic field and the film exhibits complete transparency visually, the saturation voltage being the correlating applied voltage (V_sat_) [37]. The contrast is $\mathrm{CR}=T_{\mathrm{on}}/T_{\mathrm{off}}$. Through the combination of types and amounts of LC materials and polymer monomers, higher CR can be achieved with exhibiting lower off-state transmittance.

Investigating the microscopic patterns of PDLC films was performed using scanning electron microscopy (SEM, ZEISS EVO 10). Until the LC molecules vanished, the ready PDLC was drenched in cyclohexane for two weeks at ambient temperature, and the cyclohexane solution requires daily replacement. Following soaking, the material was dried for 12 h at room temperature in a drying oven before being sputtered to gold for SEM analysis. In addition to SEM, polarized light microscopy (POM, ZEISS Axio Lab. A1 Pol) is an equally effective means of observing the microscopic morphology of films and fibers. In order to investigate the spinning time effect on fiber film formation, the prepared samples were stored in a room temperature atmosphere for 6 h, and then the morphology was observed at a size of approximately 1 cm^2^.

## 4. Conclusions

As a result, the problems with traditional PDLC devices, such as lower contrast and poor stability of performance, are improved by adding PMMA nanofiber films with uniform morphology to the liquid crystal/polymer polymeric mix. By analyzing the morphology, Vth, Vsat, and response time, it can be proven that the proportion of liquid crystal/polymer monomers empowers the advancement of polymer networks with various chamber lengths and widths, which undoubtedly influences the electro-optical properties of PDLC films. The most surprising thing is that after partial polymer networks were replaced by nanofibers, the polymer networks were affected obviously and the electro-optical properties of PDLC films were significantly optimized. After the introduction of PMMA nanofiber films into the PDLC, the CR can be increased by 100%. At the same time, the stability in extreme environments (60 °C, 95% RH, 365 nm) is substantially improved, and the outdoor service life of PDLC is significantly improved. All research results have proven that introducing polymer nanofibers into PDLC films is a highly valuable improvement, especially in enhancing both the photoelectric performance and service life of the films.

## Figures and Tables

**Table 1:** Electrospun nanofilm sample compositions.

Sample	PMMA (wt. %)	DMF (wt. %)	Electrospinning Time (min)
b1	25	75	5
b2	25	75	10
b3	25	75	15
b4	25	75	20
b5	25	75	25

**Table 2:** Sample compositions.

Sample	Weight Percentage (%)		Electrospinning Nanofilm Samples
	SLC1717	UV6301	
Group A			
A1	90.0	10.0	0
A2	80.0	20.0	0
A3	70.0	30.0	0
A4	60.0	40.0	0
A5	50.0	50.0	0
Group B			
B1	80.0	20.0	b1
B2	80.0	20.0	b2
B3	80.0	20.0	b3
B4	80.0	20.0	b4
B5	80.0	20.0	b5
Group C			
C1	90.0	10.0	b3
C2	80.0	20.0	b3
C3	70.0	30.0	b3
C4	60.0	40.0	b3
C5	50.0	50.0	b3

## Data Availability

Not applicable.

## Sample Availability

Samples of the compounds are not available from the authors.
